# Comparative Radiographic Analysis of Trochleoplasties for Patellar Luxation Correction: Inter-Observer Agreement of a Modified Osteoarthritis Scoring System

**DOI:** 10.3390/ani15111639

**Published:** 2025-06-03

**Authors:** Nikolaus Velich, Britta Vidoni, Eberhard Ludewig, Alexander Tichy, Eva Schnabl-Feichter

**Affiliations:** 1 Tierklinik St. Pölten, 3100 St Pölten, Austria; 2 Clinical Department Small Animals and Horses/Small Animal Surgery, University of Veterinary Medicine, 1210 Vienna, Austria; 3 Clinical Department of Small Animals and Horses/Diagnostic Imaging, University of Veterinary Medicine, 1210 Vienna, Austria; 4 Department Bioinformatics and Biostatistics, University of Veterinary Medicine Vienna, 1210 Vienna, Austria

**Keywords:** dog, small breed, medial patellar luxation, trochleoplasty, trochlear wedge recession, trochlear block recession, osteoarthritis scoring system

## Abstract

Patellar luxation is common in small breed dogs and is often treated surgically. This study compares the long-term outcomes of two surgical techniques, trochlear wedge recession (TWR) and trochlear block recession (TBR), for medial patellar luxation (MPL) with regard to osteoarthritis (OA) progression. High inter-observer agreement was hypothesized for the use of a modified OA scoring system and fewer OA changes in the TBR group. This study included 25 dogs (<15 kg) with grade-2 or -3 MPLs treated at the University of Veterinary Medicine Vienna (2016–2021). A total of 32 stifle joints (TWR Group n = 11, TBR Group n = 21) were evaluated pre-operatively and at least one year post-operatively using a modified OA scoring system. A statistical analysis was conducted to compare OA progression between the techniques. The inter-observer agreement was high. The OA scores increased in both groups post-operatively, with TBR demonstrating a greater progression at specific points. Overall, no significant differences were found between the techniques. These findings suggest that OA progression does not significantly differ between TWR and TBR. The modified OA scoring system has shown to be reliable for assessing OA progression after treatment. Further prospective studies with larger study populations incorporating clinical assessments are needed for a better understanding of the surgical impacts on OA development.

## Simple Summary

Patellar luxation is a common knee problem in small breed dogs, where the patella most often is displaced medially, often requiring surgery for a permanent stabilization. This study compares two surgical techniques in 25 small dogs with moderate to severe patellar luxation to see which technique led to better long-term outcomes. Radiographs were taken before and at least one year after surgery to track joint changes. Three experienced veterinarians reviewed the radiographs using a scoring system to evaluate osteoarthritis (OA) progression. The results revealed that the degree of osteoarthritis increased in both groups over time, but one group had slightly worse outcomes in some cases. Overall, no major differences were found between the two treatment options regarding the extent of osteoarthritis development. The scoring system used in this study proved to be useful for tracking OA progression after surgical treatment of patellar luxation. More research with larger groups of dogs and additional tests, such as movement analysis, are needed to better understand how surgery affects long-term outcomes.

## 1. Introduction

Patellar luxation often occurs in small dog breeds, with an increasing amount of large dog breeds being affected as well [1,2,3,4,5]. The pathophysiology behind the condition is not clearly understood, though most cases are considered developmental, with anatomical deformities leading to failure of the stifle extensor mechanism [6,7,8,9].

Patellar luxation is classified by cause (traumatic or congenital/developmental) and localization (lateral or medial), ranging in severity from grades 1 to 4 [10]. The most common type in small breed dogs is medial patellar luxation (MPL), with a significant number of dogs suffering from bilateral disease [2,3,11]. The most widely used surgical correction methods, indicated in most grade-2–3 luxations, involve tibial tuberosity transposition, frequently paired with femoral trochleoplasty and various soft tissue techniques depending on the trochlea depth [6,12,13]. For grade-4 luxations, post-operative re-luxations, or cases with severe limb deformities, additional surgical interventions such as distal femoral osteotomy (DFO) may be necessary [14,15,16,17]. Trochleoplasty techniques, which aim to deepen shallow trochlea, traditionally include trochlear sulcoplasty, trochlear chondroplasty, trochlear block recession (TBR), and trochlear wedge recession (TWR) [6,18,19]. However, recently, a new technique called semi-cylindrical recession trochleoplasty has been evaluated in a pilot study in dogs, with short-term follow-up showing similar functional outcomes between the semi-cylindrical recession trochleoplasty (SCRT) and trochlear block recession techniques regarding the rate of re-luxation and limb function [20]. Another study in cats demonstrated that SCRT combined with soft tissue reconstruction was a viable option for surgical correction of medial patellar luxation, as short-term outcomes showed only minor complications, and all patellae remained centralized. Additionally, a cadaver study comparing SCRT and TBR found that femoropatellar joint contact pressure in the SCRT group returned to levels close to those of the normal joint mechanism [21,22].

Though no empirical data are available, the most popular techniques used to correct medial patella luxation are either TBR or TWR. In a cadaver study, TBR in the extended knee joint results in increased patellar articular contact with the recessed trochlea, compared with that following TWR [23]. Consequently, the authors concluded that fewer osteoarthritic changes could be expected in canine knee joints treated with trochlear block than with trochlear wedge recession [23]. Ex vivo studies can provide an idea of how well a surgical technique works. Nevertheless, to be able to make more accurate conclusions about the actual short-term and long-term outcomes of a technique, clinical studies must be conducted.

Despite the widespread use of TBR and TWR in MPL surgery, comparative clinical data on their long-term radiographic outcomes—particularly regarding osteoarthritis progression—are lacking, making it difficult to guide evidence-based surgical decisions. Given that a comparative study of these two techniques has recently been published for a small cohort, the aim of this study was to build upon their findings and address this relevant clinical gap by evaluating whether a modified scoring system for OA changes in the knee joint is a suitable and objective method for evaluating pre- and post-operative radiographs of small dogs undergoing correction surgery for MPL [24].

A novel aspect of this study is the evaluation of inter-observer variability among clinicians with varying levels of experience. This approach provides insight into the reliability and practical applicability of the modified scoring system, which is essential for standardizing the assessment of radiographic outcomes in clinical practice. To quantify OA progression over time, we used a modified scoring system from Wessely et al. (2017) [25]. In their study, they found the intra-observer variability for this OA score to be low, which is why we decided not to repeat it in our study. As for our primary hypothesis, we expected a high inter-observer agreement between our three observers using the mentioned scoring system. Based on previous studies and clinical reasoning, we further hypothesized that TBR would lead to fewer osteoarthritic changes in the femoropatellar joint compared with those from TWR [23]. We also expected that more than 50% of cases treated for medial patellar luxation (MPL) would show increases in their overall OA scores by 5–10 points, regardless of the technique used.

## 2. Materials and Methods

This retrospective study was conducted following approval by the Ethics and Animal Protection Committee of the University of Veterinary Medicine Vienna (ETK-014/02/2021). Written consent was given by the owners of each patient included in this study.

### 2.1. Animals

The medical records of dogs that had undergone surgical correction of medial patellar luxation at least one year ago (between 2016 and 2021) at the Clinical Centre of Small Animals at the University of Veterinary Medicine Vienna were retrieved. The inclusion criteria comprised skeletally mature dogs under 15 kg with grade-2 or -3 medial patellar luxation and no other orthopedic disorders of the affected stifle joints (e.g., cruciate ligament issues). Patients were selected regardless of their breed or sex. Dogs were considered skeletally mature when a complete closure of growth plates was observed.

In total, 84 client-owned dogs that met the criteria were identified in the system. All 84 owners were contacted via telephone to take part in this study and invited for a re-examination. Finally, a total of 25 dogs were re-examined, with some being affected bilaterally, leading to a total of 32 stifle joints. Among them, 23 stifle joints showed grade-3 medial patellar luxation, with the remaining 9 showing grade-2 medial patellar luxation.

Regarding the surgical procedures, 11 stifle joints underwent trochlear wedge recession, and the remaining 21 underwent trochlear block recession. Most dogs in this study were Chihuahuas (n = 12), followed by Russkiy Toy Terriers (n = 4), mixed breed dogs (n = 3), and a variety of other small dog breeds (Maltese and Whippet, n = 2; Miniature Pinscher, Affenpinscher, Biewer Yorkshire Terrier, Yorkshire Terrier, Bolonka Zwetna, Pomeranian, and Jack Russel Terrier, n = 1 each). The numbers of female and male dogs were 21 and 11, respectively. Their mean bodyweight was 2.8 ± 3.5 kg (2.1–12.5 kg) (Supplementary Material Table S1).

### 2.2. Surgery

All surgeries were conducted by a board-certified specialist, senior staff surgeon, or resident in training. The choice of trochleoplasty was left up to the operating surgeon’s discretion, with 21 knee joints having undergone TBR, and the remaining 11 having undergone TWR. At our institution, multiple senior orthopedic surgeons perform these procedures, and the choice of technique is primarily influenced by individual surgeon preference, which is in turn shaped by their training background and mentorship. Some surgeons favor the block recession technique due to their residency training, while others prefer the wedge technique based on earlier clinical experience.

At the time of surgery, only unilateral procedures were performed. All surgeries included an additional transposition of the tibial tuberosity, followed by fixation with two Kirschner wires. Soft tissue techniques, including retinacular release or imbrication, were also performed; however, these methods were not consistently documented in the medical records and, thus, could not be formally analyzed.

### 2.3. Radiographic Examination and Scoring

Radiographs of the limbs were acquired using a stationary X-ray machine (Axicom Iconos R200, Siemens Healthineers, Erlangen, Germany). The images were recorded with a digital flat-panel detector (FDR D-EVO II C24, Fujifilm, Tokyo, Japan). The exposure settings were adjusted to achieve exposure indicator values in the range of S = 400 ± 40 doses. The mediolateral radiographs showed knee angles in the range of 85° to 95°. To obtain the caudocranial images, each dog was positioned in sternal recumbency with the affected limb extended along the long axis of the femur and parallel to the long axis of the tibia. Radiographs were taken pre-operatively and at least one year after surgery.

**Figure 1:**
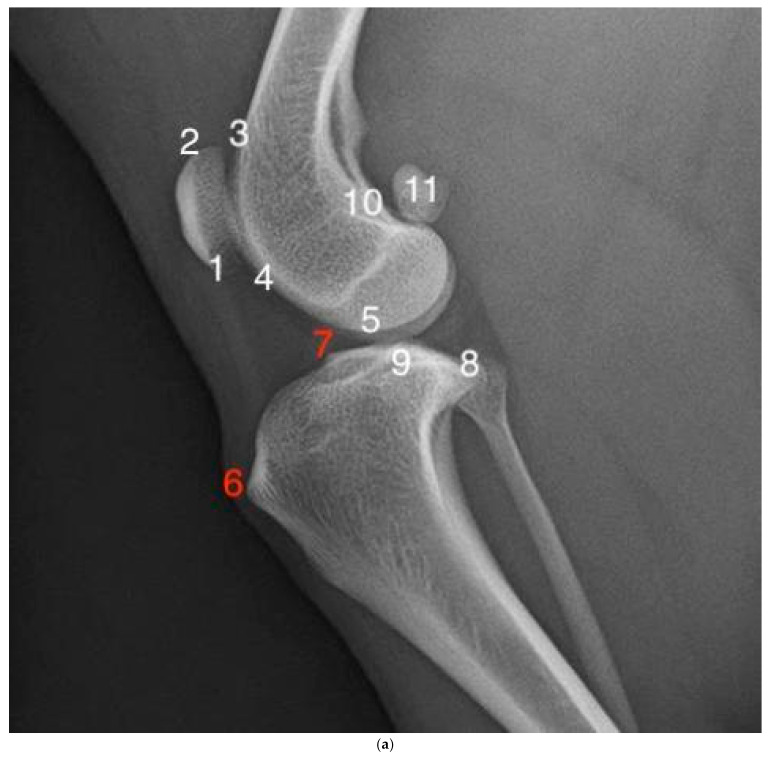

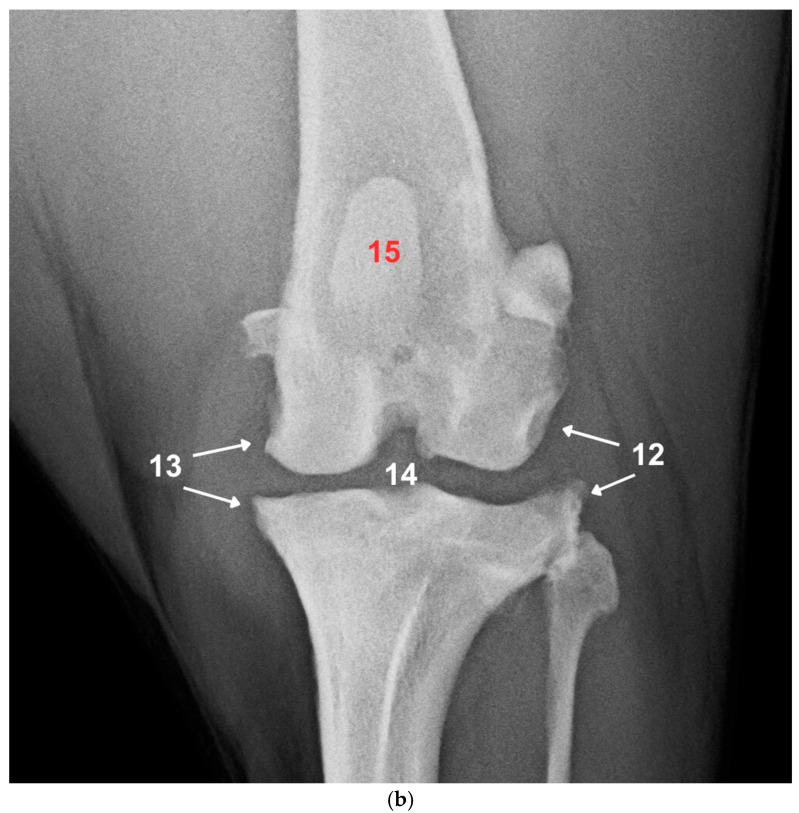
(**a**) Assessment points for the OA score in the mediolateral view. The following bone prominences were evaluated: apex of the patella (1), base of the patella (2), proximal trochlear ridge (3), distal trochlear ridge (4), femoral condyle (5), caudal aspect of the tibial plateau (8), central aspect of the tibial plateau (9), popliteal surface of the femur (10), and sesamoid bones (11). The points marked in red (6 and 7) were left out of the assessment. (**b**) Assessment points of the OA score in the caudocranial view. The following bone prominences were evaluated: lateral tibial and femoral epicondyle (12), medial tibial and femoral epicondyle (13), and intercondylar notch (14). The point marked in red (15) was left out of the assessment.

In order to evaluate whether progression of OA took place between surgery and follow-up, a modified version of the OA scoring system published by Wessely et al., in 2017, was used [25]. We modified the scoring system by removing the following three originally described areas to prevent any interference due to callus formation after tibial tuberosity transposition, implants still in situ, and double scoring: the tibial tuberosity (formerly point 6), the cranial aspect of the tibial plateau (point 7), and the patella (point 15) in the craniocaudal view (Figure 1a,b). With the remaining 12 points available for assessment, the OA scores for each individual radiograph ranged from 12 to 48 points.

To ensure consistent application of the evaluation criteria, a training session was conducted prior to the rating sessions. In this training session, the observers were introduced to the methodology and the assessment tool by use of a detailed illustrated description and stifle radiographs not part of this study (Table 1).

The radiographs were then assessed by three independent observers with varying degrees of experience and training: a board-certified radiologist (Dip ECVDI; Observer 1), a surgical intern (Observer 2), and a senior orthopedic surgeon (Observer 3).

The observers evaluated the images without reference images. Observers were blinded to the pre- or post-operative radiographs with regard to the name and age of the patient, surgical technique used (TBR or TWR), and time point relative to the date of surgery (pre- or post-operative); however, the mediolateral and craniocaudal images of the same patient were evaluated together as paired images.

### 2.4. Statistical Analysis

The changes in OA scores over time and the difference between the two groups were analyzed with a general linear model for repeated measures (GLM). The assumption of a normal distribution of the average OA scores was tested using the Shapiro–Wilk test. The effect of time between the pre- and post-operative measurements on the OA score was investigated using Pearson correlations. The inter-observer agreement was measured based on the intraclass correlation coefficient (ICC). To determine the difference in OA scores for each individual point and operational technique performed, a U-test (Mann–Whitney U Test) was used. The statistical software used to perform the data analysis was IBM SPSS v.29.

### 2.5. Use of AI in Manuscript Preparation

This manuscript benefited from the assistance of a generative AI tool (ChatGPT-4, OpenAI, version April 2024), which was used to translate from German to English, to correct spelling mistakes, and, lastly, to improve the grammar and structural coherence of the written content. All scientific content, analysis, and interpretation were developed independently by the authors.

## 3. Results

**Figure 2:**
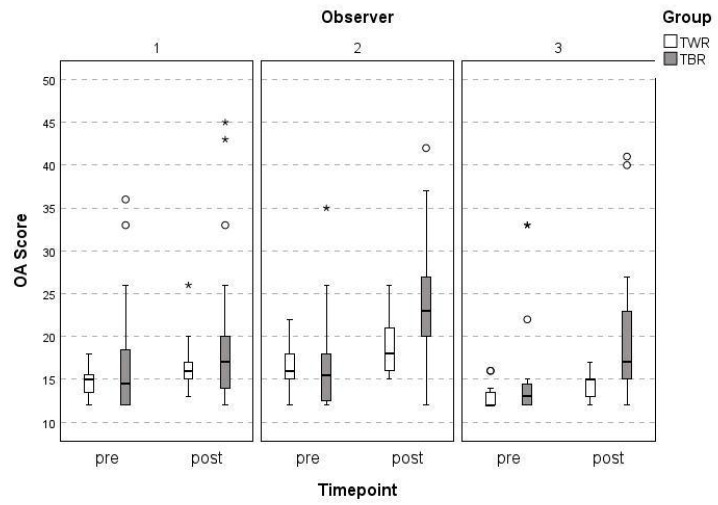
The mean OA scores of the three observers (1 = ECVDI radiologist; 2 = surgical intern; 3 = senior surgeon; TWR = trochlear wedge resection; TBR = trochlear block resection). Circle = mild outliers, * = extreme outliers.

Of the 32 stifle joints, 31 were evaluated by the three observers independently, with one evaluation excluded due to inadequate positioning. The median time of follow-up was 37 months ± 26 (14–91 months) in the TWR group and 36 months ± 29 months (13–96 months) in the TBR group. The mean overall pre- and post-operative OA scores of the TBR and TWR were 16.19 and 19.00 (Observer 1), 16.74 and 22.41 (Observer 2), and 14.52 and 17.87 (Observer 3), respectively (Table 2). The inter-observer agreement of the three observers at the two time points for each of the surgical techniques is high (ICC = 0.812 for pre-OP; ICC = 0.786 for post-OP, *p* < 0.001). However, less agreement was found when comparing the differences (increase in OA score over time) in their evaluations. The surgical intern (Observer 2) gave significantly higher overall post-operative OA scores than the board-certified radiologist (Observer 1) and the senior surgeon (Observer 3) did (ICC = 0.499, *p* < 0.001) (Figure 2).

**Figure 3:**
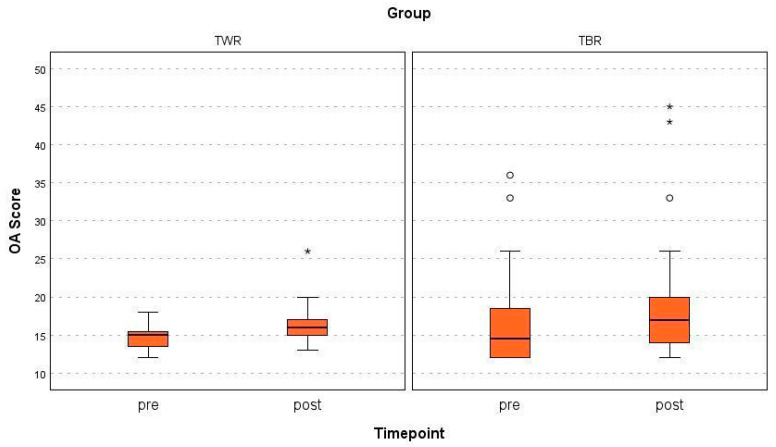
The total pre- and post-operative OA scores for both groups (TWR = trochlear wedge resection; TBR = trochlear block resection). Circle = mild outliers, * = extreme outliers.

No significant differences were found in the post-operative OA scores between wedge and block sulcoplasty (*p* = 0.126); both groups had significantly higher scores during their follow-up examinations compared with those before surgery (*p* < 0.001) (Figure 3).

The mean increase in the total OA score of all three observers was 1.9 points in the TWR group and 5.2 in the TBR group, significantly in the TBR group (*p* < 0.001) but not in the TWR group (*p* = 0.084).

The board-certified radiologist and the senior orthopedic surgeon both gave 11 out of 31 (35.4%) post-operative radiographs an OA score of ≥5 compared with their pre-operative radiographs. In contrast, the surgical intern gave 19 out of 31 (61.1%) post-operative radiographs an OA score of >5.

**Figure 4:**
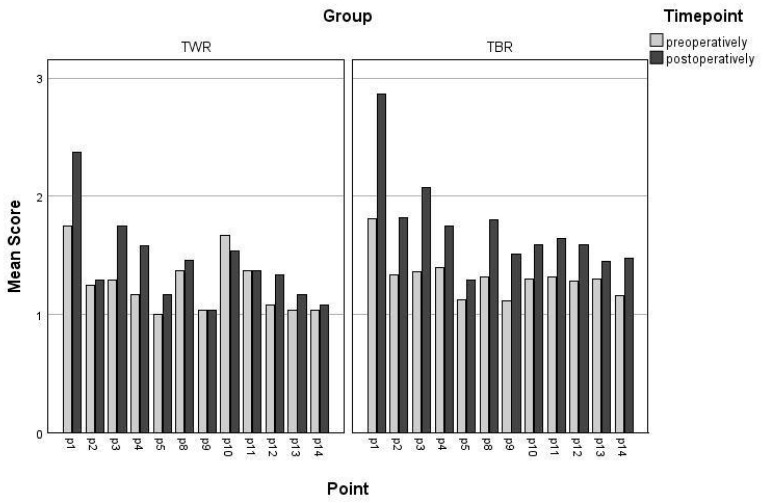
The mean pre- and post-operative OA scores for each individual bone point.

Irrespective of the surgical technique applied, no significant correlation was found between the number of months between the radiographs and the OA score. Examining each bone point individually, the central tibial plateau (point 9) in the TWR group is the only point that displayed a significant increase in the OA score during the follow-up examination (*p* = 0.03) compared with that pre-surgery (Figure 4).

## 4. Discussion

This study investigated the radiographic outcomes of trochlear block recession (TBR) and trochlear wedge recession (TWR) for the correction of medial patellar luxation (MPL). The results reveal that both techniques led to an increase in osteoarthritic changes, with TBR showing a greater mean increase in OA scores compared with that following TWR.

Our results support our primary hypothesis, with the inter-observer agreement being high at both the pre- and post-operative timepoints. We chose the scoring system by Wessely et al. for its feasibility and already validated low inter- and intra-observer variability [25]. Similarly to that study, the least experienced observer (the surgical intern) also yielded the most variable evaluations in our study, which is the reason for the lower intraclass correlation coefficient (ICC) during the follow-up examination compared with the high ICC at the initial radiographic evaluation [25]. A possible explanation for these results could be the difference in the overall experience in seeing and evaluating orthopedic radiographs, since both senior clinicians have over 20 years of experience compared with only 2 years for the surgical intern. We intentionally kept this less experienced observer to reflect the diversity of clinical evaluators and to examine whether the scoring system retained its reliability under more variable real-world conditions. While this increases inter-observer variability, our data still show high agreement among all observers, supporting the robustness of the modified OA scoring system. However, the fact that our findings mirrored those of Wessely et al., strengthens the conclusion that observer experience can influence scoring consistency. This repeated finding between the two studies suggests that future radiographic research might be improved by limiting evaluations to experienced observers only, thereby reducing variability and enhancing data reliability.

We failed to support our other hypothesis that trochlear block recession would lead to less osteoarthritic changes compared with trochlear wedge recession. Interestingly, patients with trochlear wedge recession appeared to have at least radiologically more favorable outcomes considering the mean increase in the total OA score in the TBR group during the follow-up examination after surgery.

Our final hypothesis, which posited that more than 50% of all patients would show an increase in their OA score by more than 5 points at the follow-up examination regardless of the used surgical technique, is only partially supported. Only the surgical intern evaluated over 50% of patients as showing an increase of >5 points, while the two senior observers (the senior orthopedic surgeon and the board-certified radiologist) clearly gave different results.

The reason we modified the scoring system before starting the evaluations was to provide objective data on osteophyte formation at locations that were previously unharmed, thereby excluding any bony reactions due to iatrogenic trauma during surgery. To avoid double scoring, we also removed the patella score (former point 15) from the caudocranial projection, as it is already evaluated in the lateral view. On an additional note, after testing this system on various radiographs of small dog breeds, assessing the patella in the caudocranial view was not feasible due to femur superimposition, unlike in large dog breeds, where the bigger patella leads to a much better contrast on radiographs of the knee joint.

Various studies have evaluated the short-term outcomes in dogs undergoing MPL surgery [11,26,27]. Nevertheless, only two comparable studies have clinically and radiologically evaluated the long-term outcome of dogs undergoing surgery using tibial tuberosity transposition and sulcoplasty for the treatment of medial patellar luxation [24,28]. Recent findings support the general effectiveness of both TBR and TWR, though they noted a higher risk of medial patellar re-luxation with TWR (Vodnarek et al. 2024) [24]. Their study also found that, while OA scores increased in both groups, most owners perceived a favorable outcome, suggesting that clinical satisfaction may not always correlate directly with radiographic changes. These results align with our observations of OA progression in both groups. Unfortunately, precise data on where the radiographic changes are most prominent were not recorded in either of the two studies [24,28].

In our study, however, we put particular emphasis on where the changes were most significant and revealed the complexities in the relationship between surgical technique and osteoarthritis progression. Since two different types of trochleoplasties were used, we assumed that the bone points most affected by the surgical intervention (the proximal trochlear ridge (3) and the distal trochlear ridge (4) in Figure 1a) would show greater changes over time at the follow-up examination. Interestingly, those points showed no significant difference in our study, whereas only the central tibial plateau (point 9) in the TBR group showed a significant increase during the follow-up examination. A plausible explanation as to why this was the only point with a significant change over time could be the onset of cranial cruciate disease. Since medial patellar luxation and cranial cruciate rupture share a close connection, these bony changes could mark the beginning of the degeneration of the cranial cruciate ligament [6,29].

Despite the broad follow-up intervals of 14 and 91 months, no significant correlation was found between the duration of follow-up and the OA score. This suggests that the observed OA progression was not only a function of time. Nevertheless, future studies should aim to use standardized follow-up intervals to improve comparability between cases.

Although block sulcoplasty gained widespread popularity after the publication of a cadaver study by Johnson et al, clinical and radiological data have not been sufficiently provided to support its use over wedge sulcoplasty, considering that the latter achieves favorable outcomes as well [23]. The potential benefits of the increased surface area of the articular cartilage in block sulcoplasty is one of the main reasons this technique is used. A recent study also showed a positive influence on pelvic limb alignment in patients undergoing TBR compared with that for TWR [30]. However, definitive proof for these benefits remains absent. The increased area of hyaline cartilage compared with that following TWR can have positive clinical relevance, as cartilage erosion is a potential reason for unsatisfactory clinical outcomes and progression with OA even after a technically successful MPL surgery [31,32]. In retrospect, while our study did not demonstrate such an advantage, we do not wish to dismiss the potential benefit of TBR over TWR outright. Conversely, our results should not be disregarded either. One plausible explanation for the higher OA scores observed in the TBR group could be the greater exposure of subchondral bone associated with this technique. Given the relatively small sample size in our study, however, the possibility that these findings are due to random variation cannot be excluded.

As for its clinical relevance, our results should be interpreted with caution. Although studies show a correlation between radiological changes and clinical signs of OA, namely, lameness and reduced function, some patients also suffer from degenerative joint disease, where radiographic findings do not always correlate with clinical function [33,34]. Nonetheless, by integrating radiographic scoring with observer reliability, this study offers a useful methodological approach for future clinical studies. For a detailed analysis of the long-term clinical outcomes, a prospective study design with a questionnaire for owners, clinical and orthopedic examinations of the dogs, and, most important, a force-plate analysis should be performed.

This study has several limitations inherent to its retrospective design. One key limitation is the subjective selection of the surgical technique, as the decision between trochlear block recession (TBR) and trochlear wedge recession (TWR) was based on the individual surgeon’s training and experience. This reflects real-world clinical practice but reduces reproducibility and may have influenced the surgical outcomes. Additionally, the inconsistent documentation of soft tissue procedures prevented us from analyzing their potential impact on joint function and radiographic OA progression. Observer variability was another limitation, as radiographs were assessed by clinicians with varying levels of experience. While this approach was intended to reflect clinical diversity, it may limit generalizability, despite high agreement among observers. Future studies could benefit from standardized observer training or limiting evaluations to more experienced clinicians to enhance consistency. Finally, the small sample size (31 knee joints) and wide range of follow-up durations (14–91 months) may affect interpretation of results. However, regression analysis did not identify a significant correlation between follow-up time and OA scores, suggesting that follow-up variability did not systematically bias the findings. Future research should aim to address these limitations through larger, prospective, and randomized studies with standardized follow-up intervals to validate and expand upon our findings.

## 5. Conclusions

Our study provides insight into the radiographic long-term outcomes of two surgical techniques—trochlear block recession and trochlear wedge recession—for the treatment of medial patellar luxation (MPL) in dogs. The modified OA score used to evaluate knee joints affected by MPL demonstrated reliability regarding inter-observer variability, suggesting its potential as a useful tool for objectively assessing surgical outcomes. However, as observed in the original study by Wessely et al., more experienced observers tended to produce more consistent results, indicating that future radiographic studies may benefit from involving experienced observers to reduce variability. While this study enhances our understanding of radiographic outcomes, it does not assess clinical function or long-term prognosis and, therefore, cannot establish a direct relationship between radiographic progression and patient outcomes.

## Figures and Tables

**Table 1:** OA grading system and corresponding radiographic changes.

Grade	Severity	Changes
1	No radiological signs of osteoarthritic changes	No osteophytes/enthesophytes detectable at bone points. The bone contour corresponds to normal anatomy.
2	Minor radiographic evidence of osteophytic changes	Osteophytes/enthesophytes are detectable but do not result in loss of distinctness of the bone contour.
3	Moderate radiographic evidence of osteoarthritic changes	Osteophytes/enthesophytes are clearly visible, and loss of bone contour may be present.
4	High-grade radiographic evidence of osteoarthritic changes	Osteophytes/enthesophytes extend well beyond the bone contour and may be associated with significant loss of distinctness of the bone contour.

**Table 2:** Scores of the three observers for the evaluation pre-operative and post-operative (TWR = trochlear wedge recession; TBR = trochlear block recession; SD = standard distribution).

	Observer 1				Observer 2				Observer 3			
	Pre-Operative		Post-Operative		Pre-Operative		Post-Operative		Pre-Operative		Post-Operative	
	Mean + SD	Range	Mean + SD	Range	Mean + SD	Range	Mean + SD	Range	Mean + SD	Range	Mean + SD	Range
TWR	14.7 + 1.7	12–18	16.9 + 3.5	13–20	16.6 + 3.0	12–22	18.8 + 3.6	15–26	13.0 + 1.6	12–16	14.3 + 1.6	15–26
TBR	17.0 + 6.9	12–36	18.6 + 7.6	12–45	15.4 + 6.4	12–35	23.4 + 5.3	12–42	15.4 + 6.4	12–33	18.7 + 6.6	12–41

## Data Availability

The original contributions presented in the study are included in the article, further inquiries can be directed to the corresponding author.

## Supplementary Materials

The following supporting information can be downloaded at: https://www.mdpi.com/article/10.3390/ani15111639/s1, Table S1: Overview of patients and different OA Scores (TWR = trochlear wedge recession, TBR = trochlear block recession, n.a. = not applicable).

## Abbreviations

The following abbreviations are used in this manuscript:

MPL	medial patellar luxation
TWR	trochlear wedge recession
TBR	trochlear block recession
DFO	distal femoral osteotomy
SCRT	semi-cylindrical recession trochleoplasty
OA	osteoarthritis
