# Supplementary material for: Comparative Radiographic Analysis of Trochleoplasties for Patellar Luxation Correction: Inter-Observer Agreement of a Modified Osteoarthritis Scoring System
Source: Animals (Basel). 2025 Jun 3;15(11):1639. doi: 10.3390/ani15111639 (PMC12153589; doi:10.3390/ani15111639)
Supplement: Supplementary file 1 [file animals-15-01639-s001.zip › animals-3602893-supplementary.pdf]

ID	Surgery Group	Breed	Age (years)	Bodyweight in kg	Sex	Side	Time between examinations (months)	OA-Score Observer 1 pre	OA-Score Observer 2 pre	OA-Score Observer 3 pre	OA-Score Observer 1 post	OA-Score Observer 2 post	OA-Score Observer 3 post
1	TWR	Chihuahua	7.8	2.4	female	left	65	15	18	12	20	23	16
2	TWR	Chihuahua	2.3	2.5	female	right	78	12	15	12	17	18	13
3	TWR	Rusky Toy Terrier	2.3	2.2	female	left	31	16	15	16	15	16	15
4	TWR	Miniature Pinscher	7.8	4.3	male	left	39	14	18	12	13	15	12
5	TWR	Chihuahua	1.4	2.1	female	left	67	13	13	12	16	19	12
6	TWR	Rusky Toy Terrier	2.0	2.3	male	left	18	15	21	14	17	21	14
7	TWR	Rusky Toy Terrier	2.8	2.2	female	right	36	15	16	12	26	26	15
8	TWR	Mix	2.5	5.3	male	right	91	18	22	16	15	21	17
9	TBR	Chihuahua	2.1	2.6	female	left	90	17	17	12	18	30	23
10	TBR	Chihuahua	1.2	2.8	female	left	81	n.a	n.a	n.a	n.a	n.a	n.a
11	TBR	Mix	1.0	12.5	female	right	96	12	12	12	17	21	15
12	TBR	Chihuahua	0.8	3.0	male	left	23	12	12	13	15	26	15
13	TBR	Chihuahua	1.6	2.2	female	right	61	12	17	13	14	24	13
14	TBR	Malteste	10.4	4.5	female	left	34	36	35	33	43	37	40
15	TBR	Yorkshire Terrier	6.5	2.4	female	left	62	12	15	12	19	23	17
16	TBR	Whippet	0.9	11.9	female	left	30	16	18	13	19	21	20
17	TBR	Chihuahua	3.7	7.2	male	right	86	13	15	13	23	28	25
18	TBR	Chihuahua	2.5	2.6	male	right	12	15	13	12	12	12	12
19	TBR	Rusky Toy Terrier	2.5	2.3	male	right	15	14	18	12	15	22	14
20	TBR	Jack Russel Terrier	8.0	8.0	male	left	35	13	12	12	14	19	16
21	TBR	Mix	3.0	7.0	male	left	87	15	14	12	14	20	17
22	TBR	Chihuahua	1.4	2.5	male	right	37	12	12	13	13	23	13
23	TBR	Malteste	9.3	4.5	female	right	20	33	26	33	45	42	41
24	TBR	Chihuahua	4.8	3.8	male	left	57	12	12	12	16	24	14
25	TBR	Whippet	1.3	11.4	female	right	25	14	16	15	15	18	18
26	TBR	Bullterrier	2.6	12.2	female	right	49	18	20	22	33	30	27
27	TBR	Bullterrier	4.6	12.2	female	left	19	19	15	14	26	27	24
28	TBR	Pomeranian	3.5	2.3	female	left	35	19	17	14	20	23	19
29	TWR	Bolonka Zwetna	0.9	4.9	female	left	46	13	16	12	15	16	15
30	TWR	Affenpinscher	2.6	4.3	female	left	14	16	17	13	16	15	13
31	TBR	Chihuahua	7.8	3.7	female	right	15	26	20	15	18	20	17
32	TWR	Biewer Yorkshire	2.1	2.4	female	right	18	15	12	12	16	17	15

Supplemental Material Table S1: Overview of patients and different OA Scores (TWR = trochlear wedge recession, TBR = trochlear block recession, n.a. = not applicable)
